# Enhanced Removal of Malachite Green Using Calcium-Functionalized Magnetic Biochar

**DOI:** 10.3390/ijerph19063247

**Published:** 2022-03-10

**Authors:** Pengjie Wang, Wei Chen, Rui Zhang, Yanfeng Xing

**Affiliations:** Heilongjiang Ecological Environment Monitoring Center, Harbin 150000, China; wpj_19900915@126.com (P.W.); geigei_00@163.com (R.Z.)

**Keywords:** magnetic biochar, calcium modification, malachite green, adsorption mechanism

## Abstract

To efficiently remove malachite green (MG), a novel calcium-functionalized magnetic biochar (Ca/MBC) was fabricated via a two-step pyrolysis method. Iron-containing oxides endowed the target complexes with magnetic properties, especially the chemotactic binding ability with MG, and the addition of calcium significantly changed the morphology of the material and improved its adsorption performance, especially the chemotactic binding ability with MG, which could be confirmed through FTIR, XPS, and adsorption experiments. Electrostatic adsorption, ligand exchange, and hydrogen bonding acted as essential drivers for an enhanced adsorption process, and the maximum theoretical adsorption capacity was up to 12,187.57 mg/g. Ca/MBC maintained a higher adsorption capacity at pH = 4–12, and after five adsorption–desorption cycles, the adsorption capacity and adsorption rate of MG remained at 1424.2 mg/g and 71.21%, highlighting the advantages of Ca/MBC on adsorbing MG. This study suggests that biochar can be modified by a green synthesis approach to produce calcium-functionalized magnetic biochar with excellent MG removal capacity. The synthetic material can not only remove pollutants from water but also provide an efficient way for soil remediation.

## 1. Introduction

Malachite green (MG) is a triphenylmethane-based synthetic refractory organic compound with stable chemical properties [1,2,3]. As a common water-soluble cationic dye, MG is used in paper printing and dyeing processes in the textile industry, and it is also widely used in fisheries as an antibacterial agent and antifungal agent [3,4]. However, MG and leucomalachite green (an MG metabolite) are highly carcinogenic and genotoxic, and can cause significant damage to the human body, especially the immune and reproductive systems [3,4,5,6]. Wu et al. [7] indicated that key components of soil ecosystems are adversely affected by MG and will eventually have a significant impact on the soil environment and cause ecosystem damage. Since the mid-1990s, when MG was finally proven to be toxic enough to be prohibited in seafood products for human consumption, 28 EU member states have regulated MG in the production and import of marine products [8]. Decree No. 235 of the Ministry of Agriculture of China in 2002 stipulates that MG is prohibited from use in all animal products and should not be detectable in animal food.

Numerous methods have been developed for removing MG, such as adsorption [9], membrane separation [10], flocculation/solidification [11], chemical treatment [12], and biological treatment methods [13]. Due to its low cost, adsorption is favored by researchers [3,9,14]. Biochar is the carbon-rich solid extracted from biomass that is usually pyrolyzed under anaerobic conditions. Biochar has been studied in dye-containing wastewater treatment because of its porosity, hydrophobic surface, high specific surface area, and excellent thermal stability [15]. Wu et al. [16] prepared biochar through hydrothermal carbonization and subsequent activation. The adsorption capacity of the samples for MG reached 2468 mg/g and involved pore filling, hydrogen bonding, π–π interactions, and electrostatic interactions. Furthermore, the modification of biochar to render it magnetic uses solid–liquid separation and recycling. Eltaweil’s group [17] prepared a nano zero-valent iron modified biochar magnetic composite that demonstrated an excellent MG removal capacity (515.77 mg/g) and recycling performance.

Magnetic biochar acquired from the pyrolysis of biomass and ferrates shows potential as an adsorbent, but it is lower than others. Surface modification is an attractive method to adjust the surface properties of magnetic biochar and improve its adsorption performance [18,19]. Several studies have shown that biochar improved by adsorbed metals, such as Cu [20], Mn [21], Fe [22], Si [23], Ti [24], and Mg [21], has incredible high pollutant removal performance. Calcium is an abundant natural resource, which is ecologically non-toxic, thus rendering it as an attractive modifier [14]. Xu et al. [25] investigated the effects of four calcium-based additives, Ca(H_2_PO_4_)_2_, Ca(OH)_2_, CaCO_3_, and CaO, on biomass pyrolysis and the properties of the resulting biochar. The results showed that CaO had the best performance for improving the biochar quality and gas production.

**Scheme 1:**
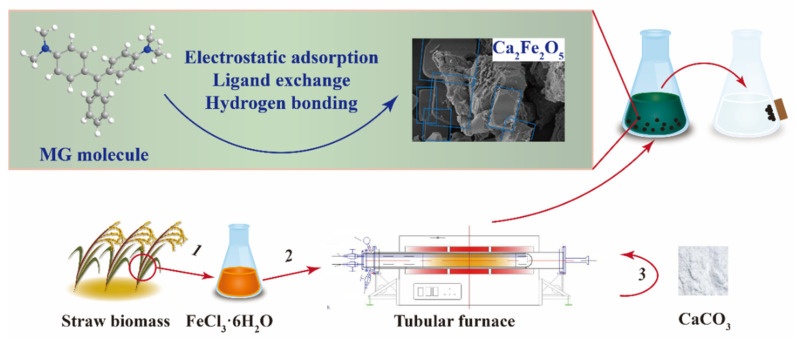
The process and mechanisms of Ca/MBC applied to MG.

Herein, to remove MG from water more efficiently, this study prepared calcium-modified biochar. To the best of our knowledge, this is the first report where porous magnetic biochar (MBC) was obtained by pyrolysis-carbonization activation, and subsequent high-temperature co-pyrolysis prepared calcium-modified magnetic porous biochar (Ca/MBC) to remove MG accordingly. Therefore, this study used adsorption isotherms, adsorption kinetics, adsorption–desorption cycles, and various characterization methods to further investigate the removal efficiency and adsorption mechanism of the adsorbents. This study provides new ideas for improving biochar and new materials for the efficient removal of organic matter from sewage (Scheme 1).

## 2. Materials and Methods

### 2.1. Materials

Rice straw was cleaned, powdered, and dried before further application. Ferric chloride hexahydrate (FeCl_3_·6H_2_O), sodium hydroxide (NaOH), calcium carbonate (CaCO_3_), anhydrous ethanol (C₂H₆O), and malachite green (C_23_H_25_C_l_N_2_) were all analytically pure and purchased from Sinopharm Chemical Reagent Co. (China). The 5 g/L stock solution of MG was prepared with deionized water. The basic properties and chemical structure of MG are shown in the Supplementary File (Figure S1).

### 2.2. Biochar Preparation and Modification

Straw biomass (10 g) was impregnated in 30 mL FeCl_3_·6H_2_O solution for 30 min with a mass ratio of 1:2, and then dried at 80 °C in an oven. The dried powder was mixed with NaOH (*w*/*w* = 1:1), heated to 700 °C in a tubular furnace at a heating rate of 10 °C/min, and then pyrolyzed for 2 h. Heating was performed under a N_2_ atmosphere with a flow rate of 200 mL/min. After cooling to room temperature, the prepared magnetic biochar was washed to a neutral pH with 500 mL of deionized water. It was then dried at 85 °C for 12 h, sieved through a 100-mesh sieve, and marked as MBC. Next, the MBC was mechanically homogeneously mixed with CaCO_3_ (*w*/*w* = 1:1), heated at a rate of 10 °C/min to 900 °C, and pyrolyzed for 2 h. Afterward, the same procedure as above was followed to obtain the sample labeled as Ca/MBC.

### 2.3. Batch Adsorption Experiments

The prepared MBC or Ca/MBC and MG aqueous solution were added to conical flasks covered with tinfoil and then shaken at 160 rpm under room temperature. The effects of the initial MG concentration, adsorption time, and initial pH on the adsorption performance of the prepared materials were analyzed. After the reaction, the Ca/MBC–liquid mixture was filtered using filter paper. The absorbance values of the collected liquids at 617 nm were measured through a UV-Vis spectrophotometer (TU-1810, Beijing, China). Specifically, the first step is the preparation of the standard solution. A certain mass of MG solid powder was weighed and dissolved in deionized water, and the original MG solution with 1 g/L concentration was prepared. After that, the standard curve was constructed. A certain amount of MG solution was weighed and diluted to the concentration range of 2, 4, 10, 25, 50, 80 and 100 mg/L. The absorbance of these six solutions was measured by a UV-spectrophotometer, and the standard curve of the MG solution was plotted according to the correspondence between concentration and absorbance. Finally, the calculations of the adsorption experiments were performed. The reaction-completed filtrate was diluted to a reasonable range, and the actual concentration of the MG solution after adsorption was calculated from the standard curve. The MG concentration in the adsorption system (*C*_0_ and *Ce*) was calculated via absorbance measurements. The equilibrium adsorption capacity *q_e_* (mg/g) and removal efficiency (*η*) were calculated using the following equations:

(1) $$q_{e}=\frac{{(c}_{0}{-c}_{e})V}{M}$$

(2) $$\eta =\frac{c_{0}{-c}_{e}\;\times 100}{c_{0}}$$

where *C*_0_ and *Ce* are the initial mass concentration of MG and its concentration at time *t*, respectively, mg/L; *V* is the volume of the solution, L; *M* is the mass of the Ca/MBC, g.

#### 2.3.1. Adsorption Kinetics and Isothermal Adsorption

The relationship between the initial MG concentration (100, 200, 400, 800, 1200, 2000 mg/L), adsorption time (10, 30, 60, 120, 180, 240 min), and adsorption capacity was investigated.

Non-linear fitting of the initial MG concentration versus adsorption was performed using the Langmuir and Freundlich models to describe the isothermal adsorption process via Equations (3) and (4), respectively. To describe the Ca/MBC adsorption kinetics for MG, non-linear fitting of the adsorption time versus adsorption amount was analyzed by using the pseudo-first-order (Equation (5)), pseudo-second-order (Equation (6)), and Elovich models (Equation (7)).

(3) $$\mathrm{Langmuir}\;\;\;\;q_{e}{=q}_{m}K_{L}C_{e}{/(1+K}_{L}C_{e})$$

(4) $$\mathrm{Freundlich}\;\;\;\;\;\;\;\;q_{e}{=K}_{F}C_{e}{}^{1/n}$$

(5) $$\mathrm{Pseudo-first-order}\;\;\;\;q_{t}{=q}_{1}{(1-e}^{{-k}_{1}t})$$

(6) $$\mathrm{Pseudo-second-order}{\;\;q}_{t}{=q}_{2}{}^{2}k_{2}{t/(1+q}_{2}k_{2}t)$$

(7) $$\mathrm{Elovich}\;\;\;\;\;q_{t}=\frac{1}{\beta }ln\left(\alpha \beta \right)-\frac{1}{\beta }ln(t)$$

where *Ce* is the equilibrium concentration of MG; *q_e_*, *q_t_*, and *q_m_* are the adsorption capacities at equilibrium, time *t*, and the maximum; *K_L_* is the Langmuir constant; *K_F_* and *n* are Freundlich constants; *t* is the reaction time; *α* and *β* are the Elovich constants; kid is the intragranular diffusion constant.

#### 2.3.2. Effect of Solution pH on the Adsorption Effect

The initial pH of the MG solution was adjusted to 2, 4, 6, 8, 10, and 12 by adding 0.1 M NaOH and 0.1 M HCl, while keeping the other conditions the same as above.

#### 2.3.3. Regeneration of Ca/MBC

The recoverability of Ca/MBC was verified via five adsorption–desorption cycles, in which Ca/MBC loaded with MG was desorbed in 100 mL anhydrous ethanol for 30 min ultrasonication. The regenerated samples were separated by magnets and dried at 80 °C [26].

### 2.4. Characterization

Scanning electron microscopy (SEM) was utilized to obtain information about the surface morphology (JSM-7500F JEOL, Tokyo, Japan). A transmission electron microscope (TEM) was utilized to obtain information about the surface morphology (Tecnai G2 F20 S-TWIN, Hillsboro, OR, USA). Before performing the SEM measurement, the samples were ground with a mortar and pestle for 5 min, then dipped into the powder using toothpicks, stuck on the conductive adhesive, and the sample surface sprayed evenly with gold using an ion sputtering coater to enhance its conductivity. Before the TEM measurement, the ground sample was dispersed in ethanol solution for more than 30 min with ultrasonic shaking, and the mixed solution was dropped onto an ultra-thin carbon grid, then placed on the sample stage for testing; X-ray diffraction (XRD) measurement was carried out using Cu Kα radiation at 40 kV and 30 mA over a range of 19–90° 2θ at 5°/min (D8, Rigaku, Tokyo, Japan). XRD patterns were processed by OriginPro-2016 peak analysis to find the 2θ and intensity ratio of each peak. Peak assignment and mineral identification were performed using the ICDD database with the 2θ position and intensity ratio of peaks of each mineral phase. The surface properties of MBC, Ca/MBC, and Ca/MBC after MG adsorption were obtained using Fourier-transform infrared (FT-IR) spectroscopy (Nicolet 460, Thermo Fisher, Waltham, MA, USA). The samples were mixed with potassium bromide powder at the mass ratio of 1:100 and milled over 5 min. Following the pressing, the sample was put into the FTIR instrument after ensuring the light transmission. Following the compression of the film poles to ensure light transmission and placement into the FTIR instrument, X-ray photoelectron spectroscopy (XPS) was performed using Al Kα excitation (Escalab 250Xi, Thermo Fisher, Waltham, MA, USA) to characterize the elemental composition of Ca/MBC before and after MG adsorption, the surface within a thickness of 10–100 nm, and the binding pattern of each element, where the detected binding energy was corrected using the C 1*s* (248.8 eV) standard peak.

## 3. Results and Discussion

### 3.1. Characterization of Samples

**Figure 1:**
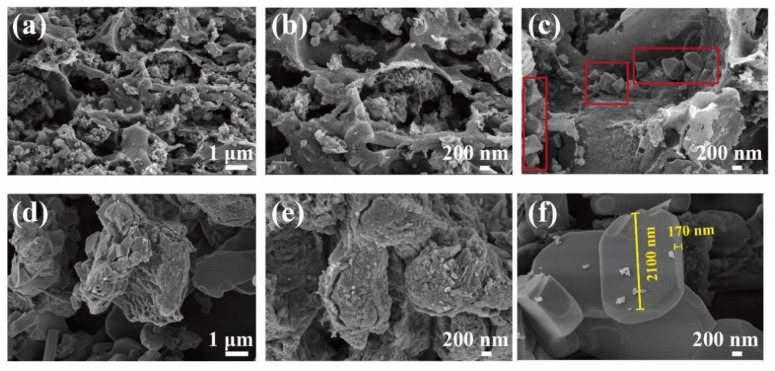
SEM images of MBC (**a**–**c**) and Ca/MBC (**d**–**f**).

**Figure 2:**
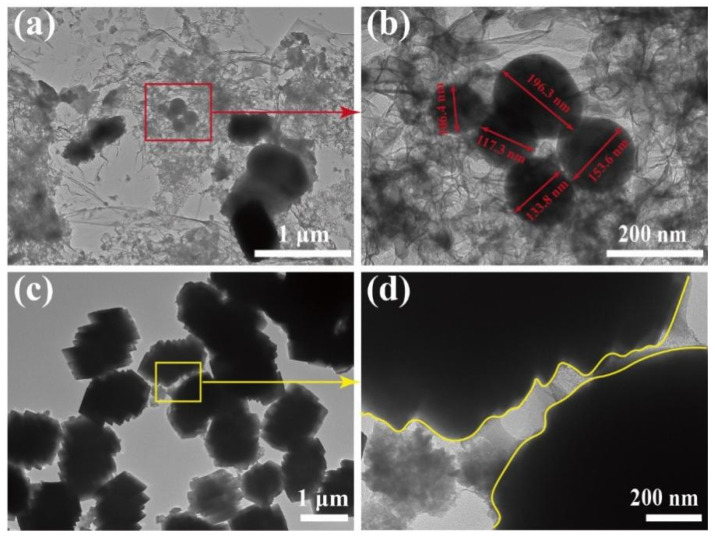
TEM images of MBC (**a**,**b**) and Ca/MBC (**c**,**d**).

The morphology of Ca/MBC was analyzed by SEM and TEM. The SEM images of the as-prepared samples at different magnifications are shown in Figure 1. The surfaces of MBC and Ca/MBC showed irregular fluffy structures with pores (Figure 1a,b), indicating that the glycosidic linkages in cellulose and hemicellulose were hydrolyzed with KOH. In contrast, the aryl linkages in lignin were cleaved (Equations (8)–(11)) [27]. The active sites on the surface of MBC and Ca/MBC may facilitate the adsorption of MG macromolecules. Intriguingly, diamond-shaped blocks were observed that adhered to the surface of MBC (Figure 1c), while Ca/MBC mainly consisted of regular clusters of rectangular-shaped substances (Figure 1f). Further structural features of MBC and Ca/MBC were clearly verified through TEM (Figure 2). Spherical substances attached to the MBC with particle sizes within 100–200 nm, and Ca/MBC was composed of biochar and large-sized regular substances that were all in concordance with the SEM results (Figure 1). To determine the qualitative components of the above observed structures, the samples were further characterized and analyzed using XRD [28].

**Figure 3:**
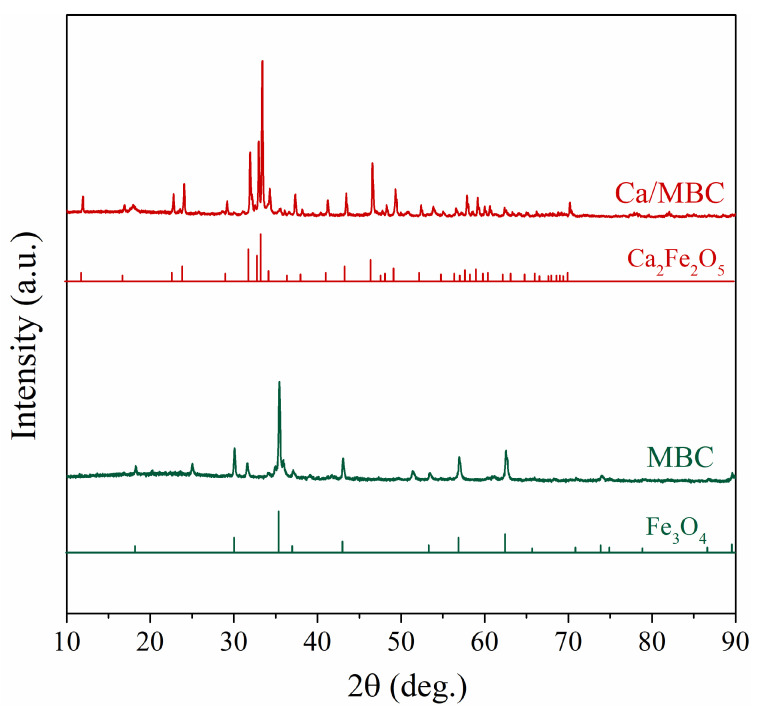
XRD spectra of BC, MBC, and Ca/MBC.

To further explore the structure of Ca/MBC, XRD analysis was performed. The XRD pattern of MBC (Figure 3) contains a series of characteristic peaks corresponding to the (111), (220), (311), (222), (400), (422), (511), (440), (662), (642), and (731) planes belonging to Fe_3_O_4_ (magnetite ICDD card PDF#00-019-0629). The pyrolysis reaction between biochar and ferrate involves the reaction pathways shown in Equations (12)–(14).There were numerous new peaks for Ca/MBC corresponding to the (020), (011), (101), (030), (131), (002), (200), (141), (051), (112), (122), (231), (161), (202), (152), (222), (062), (033), (181), (252), (143), (341), (082), (262), and (282) planes of Ca_2_Fe_2_O_5_ (calcium iron oxide; ICDD card PDF#00-018-0286), besides containing some typical MBC characteristic peaks. Combined with the SEM and TEM images, the “rhombic or spherical” substance attached to the surface of MBC may be Fe_3_O_4_, and the ‘rectangular’-like substance on the surface of Ca/MBC may be Ca_2_Fe_2_O_5_.

(8) $$6\mathrm{KOH}+2C\;\to {\;2K+2K}_{2}{\mathrm{CO}}_{3}{+3H}_{2}$$

(9) $$K_{2}{\mathrm{CO}}_{3}\;\to {\;K}_{2}{O+\mathrm{CO}}_{2}$$

(10) $$K_{2}{\mathrm{CO}}_{3}+2C\;\to \;2K+3\mathrm{CO}$$

(11) $$K_{2}O+C\;\to \;2K+\mathrm{CO}$$

(12) $${C+4\mathrm{FeCl}}_{3}{+10H}_{2}O\;\overset{∆}{\to }\;{4\mathrm{FeCl}}_{2}\cdot {2H}_{2}O+4\mathrm{HCl}↑{+\mathrm{CO}}_{2}↑$$

(13) $${\mathrm{FeCl}}_{2}\cdot {2H}_{2}{O+2H}_{2}O\;\overset{∆}{\to }{\;\mathrm{FeCl}}_{2}\cdot {4H}_{2}O$$

(14) $${\mathrm{Fe}}^{2+}{+\mathrm{Fe}}^{3+}{+8\mathrm{OH}}^{-}\;\overset{∆}{\to }\;{\mathrm{Fe}}_{3}O_{4}{+4H}_{2}O$$

**Figure 4:**
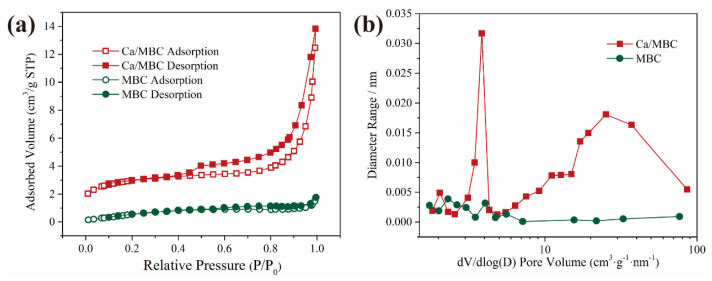
N_2_ adsorption-desorption isotherms (**a**) and pore size distributions (**b**) of MBC and Ca/MBC.

Moreover, N_2_ adsorption–desorption isotherms were obtained to obtain the specific surface area and pore characteristics information. The textural properties of MBC and Ca/MBC are reported in Figure 4 and Table S1. The N_2_ adsorption–desorption isotherms of MBC and Ca/MBC displayed a typical type IV curve with H3-type hysteresis loops, indicating that the initial monolayer–multilayer adsorption occurred in the same path as the corresponding part of the type II isotherm (Figure 4). The absence of obvious saturation adsorption plateaus in the H3-type hysteresis loop isotherm indicated an irregular structure, which was consistent with the SEM and the TEM morphology mentioned above. The surface areas (SSAs) of MBC and Ca/MBC, as shown in Table S1, were found to be significantly lower, especially for Ca/MBC, with 9.555 and 2.779, respectively. This could be accounted for by the blockage of biochar pores by Fe_3_O_4_ or Ca_2_Fe_2_O_5_ generated during pyrolysis, and also indirectly implies that with Ca/MBC as the adsorbent, chemisorption is most likely the main mechanism. Both MBC and Ca/MBC exhibited type IV adsorption–desorption isotherms, showing a wide hysteresis loop, indicating the mesoporous character of materials (Figure 4). This is consistent with the pore sizes (PS) reported in Table S1 for MBC and Ca/MBC, namely 12.684 and 4.677 nm, respectively [29,30].

### 3.2. Adsorption Study

#### 3.2.1. Effect of MBC and Ca/MBC on MG Adsorption

**Figure 5:**
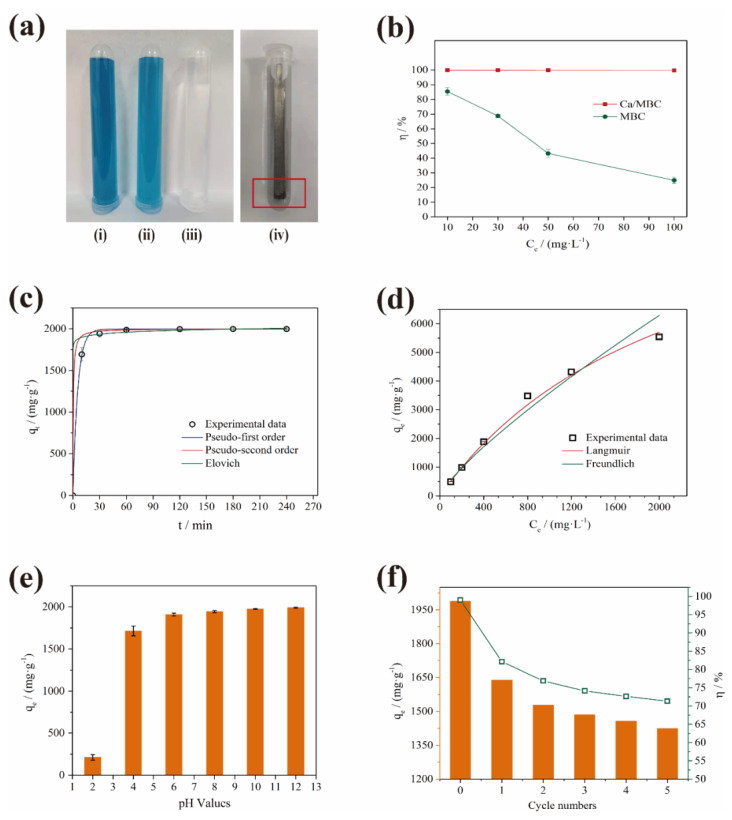
(**a**) Effect of MBC and Ca/MBC adsorption and magnetic recovery on MG: (i) 50 mg/L MG; (ii) 50 mg/L MG + MBC; (iii) 50 mg/L MG + Ca/MBC; (iv) 50 mg/L MG + MBC + magnet rod; (**b**) Comparison of MBC and Ca/MBC sorption rates on MG; (**c**) Isothermal adsorption fitting curve; (**d**) Adsorption kinetic fitting curve; (**e**) Effect of initial pH on MG adsorption by Ca/MBC; (**f**) Effect of Ca/MBC adsorption on MG after 5 times adsorption-desorption.

Adsorption capacity is the main index for evaluating the performance of an adsorbent. As shown in Figure 5a, the adsorption capacity of Ca/MBC on MG was much higher than that of MBC. The magnetic nature of Ca/MBC allowed it to be completely recovered from the MG solution, thus realizing the efficient adsorption and separation of MG. Specifically, the removal efficiency of MBC declined continuously when the sample dosage was 0.2 g/L, and the MG concentration was in the range of 10–100 mg/L. When the MG concentration was 100 mg/L, the removal rates of MG by MBC decreased to 24.92%, but the removal rates of MG by Ca/MBC were higher than 99.69% (Figure 5b).

#### 3.2.2. Adsorption Kinetics and Isotherms

Mononuclear, binuclear, and heterogeneous surface chemisorption in the solid solution system is described by the pseudo-first-order kinetics, pseudo-second-order kinetics, and Elovich models [31]. The nonlinear fitting curves and correlation coefficients *R*^2^ show that the adsorption process was consistent with the pseudo-first-order kinetics, pseudo-second-order kinetics, and Elovich models (Figure 5c and Table S2). In other words, the process of MG adsorption on Ca/MBC involved electron sharing or exchange, resulting in the formation of chemical bonds and van der Waals forces, physical adsorption, and chemical adsorption [32]. It also indicated that the adsorption rate was controlled by chemical adsorption [33]. At the same time, surface activation–deactivation and physical diffusion occurred at the interface between Ca/MBC and MG. In the Elovich fitting parameters, *α* (3.83 × 10^24^) was much greater than *β* (0.029), which indicated that the adsorption rate of MG by Ca/MBC was much higher than the desorption rate [31]. That is, the adsorption process of MG on Ca/MBC mainly involved physical adsorption, chemical adsorption, and heterogeneous diffusion at the solid–liquid interface [34].

It can be seen from Figure 5d and Table S3 that the adsorption process of Ca/MBC was better described by the Langmuir (*R*^2^ = 0.997) isothermal model than the Freundlich model (*R*^2^ = 0.974), which is consistent with previous results [35]. The Langmuir model assumes that adsorption occurs mainly via monolayer adsorption and that the surface energy of the adsorbent is uniform [36,37]. The Freundlich model assumes that adsorption occurs on heterogeneous surfaces, which is suitable for the adsorption of multi-molecular layers [38]. This shows that the adsorption of MG on Ca/MBC occurred through monolayer adsorption. The maximum theoretical adsorption capacity calculated from the Langmuir isothermal model reached 12,187.57 mg/g. Compared with several results that have been reported in the literature in the last three years (Table 1), the adsorption performance of MG by Ca/MBC is outstanding.

#### 3.2.3. Effect of pH on Adsorption

The pH of the MG solution may directly affect the surface charge of Ca/MBC, the electrostatic interactions between the adsorbent and adsorbate, and the ionization degree of pollutants [26,39]. It can be seen from Figure 5e that when the pH of the solution was 2, the adsorption uptake of Ca/MBC for MG was only 210.58 ± 32.75 mg/g. This was due to the co-existence of massive H_3_O^+^, H^+^ ions and MG in the solution. The surface protonation of Ca/MBC caused a positive charge increase, which electrostatically repelled the positively charged MG. When the pH increased to 6, the adsorption capacity of Ca/MBC for MG increased to 1908.36 ± 15.97 mg/g, which was a nine-fold increase. This shows that there is an electrostatic effect for the adsorption of MG by Ca/MBC. From pH 6–12, the adsorption of MG by Ca/MBC did not change significantly, which shows that the electrostatic effect was not the only mechanism [40]. Additionally, minor fading was observed under strong alkaline conditions, but the variation of MG adsorption on Ca/MBC in the pH range of 6–8 versus pH 10–12 was slight, and the theoretical maximum adsorption capacity was much larger than the experimental design adsorption capacity of this group, which means that the fading of MG in a strong alkaline environment in this study did not intrinsically interfere with the experimental results. Notably, a high adsorption capacity was observed in the solution pH range of 4–12, which highlights the advantages of using Ca/MBC to adsorb MG in this pH range.

#### 3.2.4. Effect of Recycling

Cyclic adsorption is an effective way to achieve Ca/MBC sustainability and reduce costs. Ca/MBC is magnetic, which promotes its recovery. As shown in Figure 5f, the adsorption effect of Ca/MBC on MG after desorption was weakened significantly compared with Ca/MBC, while the adsorption capacity and adsorption rate decreased from 1988.7 mg/g and 99.44% to 1639.3 mg/g and 81.97%, respectively. This may be due to the inactivation of some adsorption sites on the surface of Ca/MBC after adsorption–desorption. Upon increasing the number of adsorption–desorption cycles, the adsorption capacity of Ca/MBC for MG decreased gradually, but the decline was much gentler compared with the previous cycle. After five adsorption–desorption cycles, the adsorption capacity and adsorption rate of MG by Ca/MBC remained at 1424.2 mg/g and 71.21%, respectively.

### 3.3. Adsorption Mechanisms

**Figure 6:**
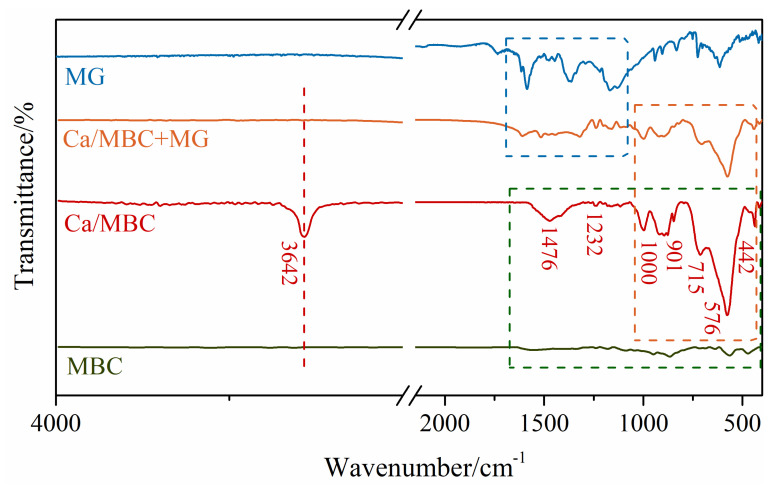
FT-IR spectra of MBC and Ca/MBC before and after MG adsorption.

As shown in Figure 6, the number and variety of functional groups on the surface of MBC were lower than those of Ca/MBC, which explains the reason that the adsorption amount was substantially lower than Ca/MBC. The peak intensity of numerous functional groups weakened after MG adsorbed onto Ca/MBC, suggesting that a series of chemical reactions occurred between the surface functional groups and MG. This supports the conclusion in Section 3.2.2 that chemisorption was involved in the adsorption process. Specifically, the O-H peak at 3642 cm^−1^ disappeared after the adsorption of MG by Ca/MBC, which indicates that the -OH groups on the surface of Ca/MBC combined with the N atoms in the MG molecule via hydrogen bonding, which increased the adsorption force [31]. Meanwhile, the peak of the spectrum after MG adsorption on Ca/MBC at 1700–1000 cm^−1^ was highly consistent with MG and only slightly shifted, which further proved the high removal ability of Ca/MBC for MG.

**Figure 7:**
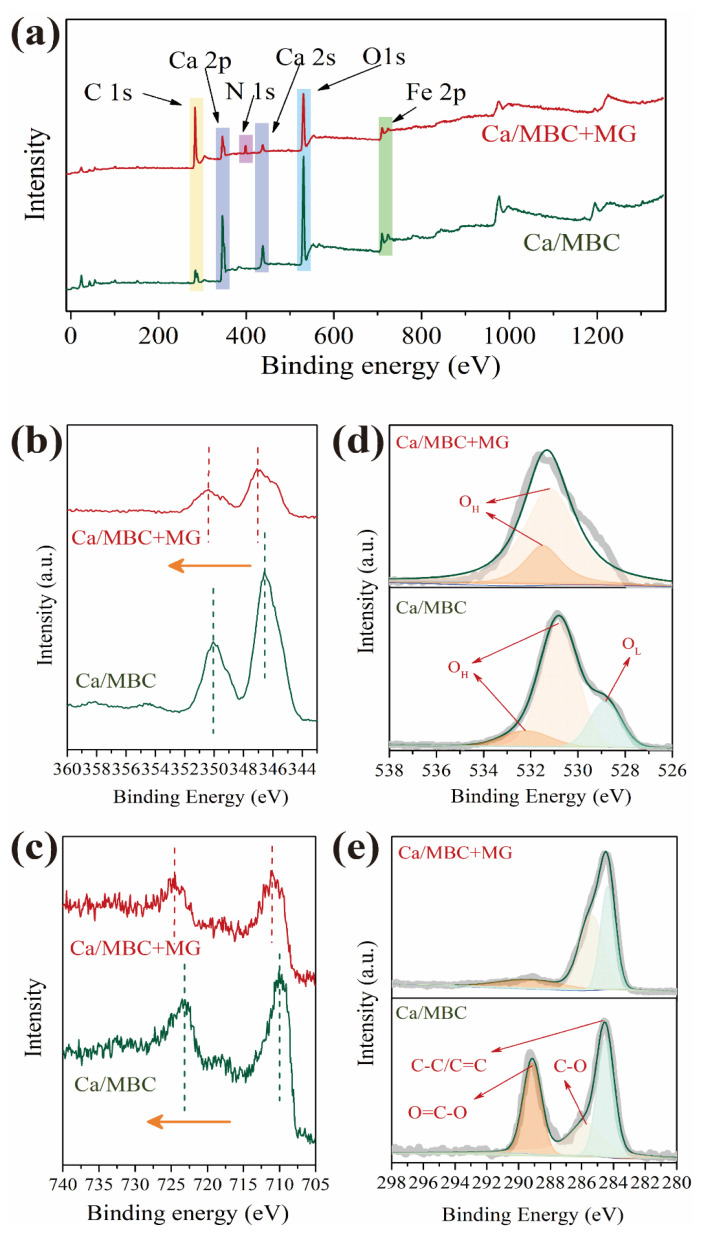
(**a**) XPS spectra of Ca/MBC before and after MG adsorption, (**b**) Ca 2*p*, (**c**) Fe 2*p*, (**d**) O 1*s*, (**e**) C 1*s*.

The XPS spectra of Ca/MBC before and after adsorbing MG are shown in Figure 7a. After MG adsorption, N 1*s* peak appeared at a binding energy of about 398.4 eV, indicating that MG was loaded on Ca/MBC. The Ca 2*p* peak of Ca/MBC had no significant shift before or after adsorbing MG, but it underwent a significant shift towards a higher binding energy, which indicates that the Ca binding state changed (Figure 7b). Because the original large number of anions in Ca/MBC formed bonds with Ca^2+^, the electron cloud density of the two structures was biased toward the anions. When MG combined with Ca^2+^, MG was more negative, leading to an increase in the binding energy of Ca 2*p* [41]. Consistent with the Ca 2*p* peak, the Fe 2*p* peak shifted towards a higher binding energy after adsorbing MG significantly (Figure 7c). The peaks at 289 eV (O=C-O) and 285 eV (C-O) decreased substantially after MG adsorption by Ca/MBC, indicating that MG replaced many carbonates during MG removal (Figure 7d).

It can be further inferred that ligand exchange occurred between Ca/MBC and MG. Figure 7e shows the two different forms of oxidation—lattice oxygen (O_L_) and hydroxyl oxygen (O_H_)—corresponding to Ca/MB before and after MG adsorption. O_H_ changed slightly after adsorption and was mainly related to Ca-OH and Fe-OH. On the other hand, a large proportion of O_L_ disappeared after MG adsorption by Ca/MBC, which further demonstrates that MG may induce lattice changes, indicating that the adsorption process was dominated by chemisorption [42].

## 4. Conclusions

In this study, calcium-modified magnetic porous biochar composites (Ca/MBC) were prepared by a two-step impregnation–pyrolysis method. The structure composition and surface functional group numbers of the calcium/iron modified biochar were optimized. Adsorption experiments and various characterization results of Ca/MBC before and after adsorbing MG showed that the adsorption mechanism mainly involved electrostatic adsorption, ligand exchange, and hydrogen bonding. The stable performance within a wide pH range (4–12) and its remarkable reusability show that this calcium-modified magnetic biochar is a potential sorbent for treating organic contaminated wastewater.

## Figures and Tables

**Table 1:** Comparison of MG adsorption capacities on various adsorbents.

Adsorbents	Adsorption Mechanisms	Adsorption Capacity	Ref.
Litchi peel biochar	Hydrogen bonding P-p interaction Pore filling Electrostatic interactions	2468 mg/g	[16]
Activated wakame biochar material	Physical adsorption Chemical adsorption	4066.96 mg/g	[9]
Kitchen waste mixed-base biochar	Physical adsorption Electrostatic Interaction Ion exchange Void filling	7434 mg/g	[31]
Calcium-functionalized magnetic biochar	Electrostatic adsorption Ligand exchange Hydrogen bonding	12,187.57 mg/g	This study

## Data Availability

The data presented in this study are available on request from the corresponding author.

## Supplementary Materials

The following supporting information can be downloaded at: https://www.mdpi.com/article/10.3390/ijerph19063247/s1, Figure S1: SEM images of MBC (a,b) and Ca/MBC (c,d); Table S1: Surface Area, Pore Volume, and Pore Size of MBC and Ca/MBC; Table S2: Adsorption kinetic fitting parameters; Table S3: Adsorption isotherm fitting parameters.

## References

[1] Kooravand M., Asadpour S., Haddadi H., Farhadian S. (2020). An insight into the interaction between malachite green oxalate with human serum albumin: Molecular dynamic simulation and spectroscopic approaches. J. Hazard. Mater..

[2] Lu J., Zhou Y., Lei J., Ao Z.M., Zhou Y. (2020). Fe_3_O_4_/graphene aerogels: A stable and efficient persulfate activator for the rapid degradation of malachite green. Chemosphere.

[3] Renita A.A., Vardhan K.H., Kumar P.S., Ngueagni P.T., Abilarasu A., Nath S., Kumari P., Saravanan R. (2021). Effective removal of malachite green dye from aqueous solution in hybrid system utilizing agricultural waste as particle electrodes. Chemosphere.

[4] Aracier E.D., Urucu O.A., Akmaki E. (2021). Imidazole modified acrylate-containing photocured hydrogels for the efficient removal of malachite green dye from aqueous solutions. J. Appl. Polymer Sci..

[5] Wei W., Pang Y., Lim S., Wong K.H., Lai C., Abdullah A.Z. (2021). Enhancement of photocatalytic degradation of Malachite Green using iron doped titanium dioxide loaded on oil palm empty fruit bunch-derived activated carbon. Chemosphere.

[6] Zhang Q., Cheng T., Lin Q., Fang C. (2021). Facile preparation of robust dual MgO-loaded carbon foam as an efficient adsorbent for malachite green removal. Environ. Res..

[7] Gopinathan R., Kanhere J., Banerjee J. (2015). Effect of malachite green toxicity on non target soil organisms. Chemosphere.

[8] Eric V., Melaine B., Marie-Pierre C., Pierrick C., Marie-Pierre F., Régine F., Murielle G., Sophie G., Dominique H.-P., Michel L. (2015). The monitoring of triphenylmethane dyes in aquaculture products through the European union network of official control laboratories. J. AOAC Int..

[9] Yao X., Ji L., Guo J., Ge S., Lu W., Chen Y., Cai L., Wang Y., Song W. (2020). An abundant porous biochar material derived from wakame (*Undaria pinnatifida*) with high adsorption performance for three organic dyes. Bioresour. Technol..

[10] Yin Y., Li C., Song C., Tao P., Sun M., Pan Z., Wang T., Shao M. (2016). The design of coal-based carbon membrane coupled with the electric field and its application on the treatment of malachite green (mg) aqueous solution. Colloid. Surf. A.

[11] Mu J., Wang D., Yang G., Cui X., Yang Q. (2019). Preparation and characterization of a substitute for ruditapes philippinarum conglutination mud as a natural bioflocculant. Bioresour. Technol..

[12] Yulizar Y., Apriandanu D., Ashna R.I. (2020). La_2_CuO_4_-decorated ZnO nanoparticles with improved photocatalytic activity for malachite green degradation. Chem. Phys. Lett..

[13] Han S., Han W., Chen J., Sun Y., Zhao G. (2020). Bioremediation of malachite green by cyanobacterium synechococcus elongatus PCC 7942 engineered with a triphenylmethane reductase gene. Appl. Microbiol. Biotechnol..

[14] Cao H., Wu X., Syed-Hassan S., Zhang S., Mood S.H., Milan Y.J., Garcia-Perez M. (2020). Characteristics and mechanisms of phosphorous adsorption by rape straw-derived biochar functionalized with calcium from eggshell. Bioresour. Technol..

[15] Murthy T.P.K., Gowrishankar B.S., Prabha M.N.C., Kruthi M., Krishna R.H. (2019). Studies on batch adsorptive removal of malachite green from synthetic wastewater using acid treated coffee husk: Equilibrium, kinetics and thermodynamic studies. Microchem. J..

[16] Wu J., Yang J., Feng P., Huang G., Xu C., Lin B. (2020). High-efficiency removal of dyes from wastewater by fully recycling litchi peel biochar. Chemosphere.

[17] Eltaweil A., Mohamed H.A., El-Monaem E., El-Subruiti G.M. (2020). Mesoporous magnetic biochar composite for enhanced adsorption of malachite green dye: Characterization, adsorption kinetics, thermodynamics and isotherms. Adv. Powder Technol..

[18] Mahanty B., Mondal S. (2021). Synthesis of magnetic biochar using agricultural waste for the separation of Cr(VI) from aqueous solution. Arab J. Sci. Eng..

[19] Agrafioti E., Kalderis D., Diamadopoulos E. (2014). Arsenic and chromium removal from water using biochars derived from rice husk, organic solid wastes and sewage sludge. J. Environ. Manag..

[20] Imran M., Iqbal M.M., Iqbal J., Shah N.S., Khan Z.U.H., Murtaza B., Amjad M., Ali S., Rizwan M. (2021). Synthesis, characterization and application of novel MnO and CuO impregnated biochar composites to sequester arsenic (As) from water: Modeling, thermodynamics and reusability. J. Hazard. Mater..

[21] Wan S., Lin J., Tao W., Yang Y., Li Y., He F. (2019). Enhanced fluoride removal from water by nanoporous biochar-supported magnesium oxide. Ind. Eng. Chem. Res..

[22] Su C., Wang S., Zhou Z., Wang H., Xie X., Yang Y., Feng Y., Liu W., Liu P. (2021). Chemical processes of Cr(VI) removal by Fe-modified biochar under aerobic and anaerobic conditions and mechanism characterization under aerobic conditions using synchrotron-related techniques. Sci. Total Environ..

[23] Liu J., Cheng W., Yang X., Bao Y. (2019). Modification of biochar with silicon by one-step sintering and understanding of adsorption mechanism on copper ions. Sci. Total Environ..

[24] Luo M., Lin H., He Y., Li B., Dong Y., Wang L. (2019). Efficient simultaneous removal of cadmium and arsenic in aqueous solution by titanium-modified ultrasonic biochar. Bioresource Technol..

[25] Xu Y., Qu W., Sun B., Peng K., Zhang X., Xu J., Gao F., Yan Y., Bai T. (2021). Effects of added calcium-based additives on swine manure derived biochar characteristics and heavy metals immobilization. Waste Manag..

[26] Zhang P., O’Connor D., Wang Y., Jiang L., Xia T., Wang L., Tsang D.C.W., Ok Y.S., Hou D. (2020). A green biochar/iron oxide composite for methylene blue removal. J. Hazard. Mater..

[27] Choudhary M., Kumar R., Neogi S. (2020). Activated biochar derived from Opuntia ficus-indica for the efficient adsorption of malachite green dye, Cu^2+^ and Ni^2+^ from water. J. Hazard. Mater..

[28] Sewu D.D., Lee D.S., Tran H.N., Woo S.H. (2019). Effect of bentonite-mineral co-pyrolysis with macroalgae on physicochemical property and dye uptake capacity of bentonite/biochar composite. J. Taiwan Inst. Chem. Eng..

[29] Chen H.R., Tsai W.T., Chou T.C., Tsai C.Y., Chang Y.M., Kuo K.C. (2011). Novel Preparation of Bamboo Biochar and Its Application on Cationic Dye Removal. J. Biobased Mater. Bioenergy.

[30] Zazycki M.A., Borba P.A., Silva R.N.F., Peres E.C., Perondi D., Collazzo G.C., Dotto G.L. (2019). Chitin derived biochar as an alternative adsorbent to treat colored effluents containing methyl violet dye. Adv. Powder Technol..

[31] Lan Y., Wang H., Li X., Jin S., Zhang Y. (2020). The Absorption of Kitchen Waste Mixed-base Biochar on Malachite Green. Chem. Lett..

[32] Meili L., Godoy R.P.S., Soletti J.I., Carvalho S.H.V., Ribeiro L.M.O., Silva M.G.C., Vieira M.G.A., Gimenes M.L. (2019). Cassava (*Manihot esculenta* Crantz) stump biochar: Physical/chemical characteristics and dye affinity. Chem. Eng. Commun..

[33] Bharti V., Vikrant K., Goswami M., Tiwari H., Sonwani R.K., Lee J., Tsang D.C.W., Kim K.H., Saeed M., Kumar S. (2019). Biodegradation of methylene blue dye in a batch and continuous mode using biochar as packing media. Environ. Res..

[34] Saeed A.A.H., Harun N.Y., Sufian S., Siyal A.A., Zulfiqar M., Bilad M.R., Vagananthan A., Al-Fakih A., Ghaleb A.A.S., Almahbashi N. (2020). Eucheuma cottonii Seaweed-Based Biochar for Adsorption of Methylene Blue Dye. Sustainability.

[35] Gokulan R., Prabhu G.G., Jegan J. (2019). Remediation of complex remazol effluent using biochar derived from green seaweed biomass. Int. J. Phytoremediat..

[36] Chahinez H.O., Abdelkader O., Leila Y., Tran H.N. (2020). One-stage preparation of palm petiole-derived biochar: Characterization and application for adsorption of crystal violet dye in water. Environ. Technol. Innov..

[37] Amin M.T., Alazba A.A., Shafiq M. (2019). Comparative study for adsorption of methylene blue dye on biochar derived from orange peel and banana biomass in aqueous solutions. Environ. Monit. Assess..

[38] Seenuvasan M., Malar C.G., Carter R., Praveen S. (2021). Magnetite embedded biochar as nano-sorbent for effective adsorption of textile dye. Latin Am. Appl. Res..

[39] Jegan J., Praveen S., Kumar B.M., Pushpa T.B., Gokulan R. (2021). Box-Behnken experimental design for the optimization of Basic Violet 03 dye removal by groundnut shell derived biochar. Desalin. Water Treat..

[40] Saravanan P., Josephraj J., Thillainayagam B.P., Ravindiran G. (2021). Evaluation of the adsorptive removal of cationic dyes by greening biochar derived from agricultural bio-waste of rice husk. Biomass. Convers. Bio..

[41] Wu D., Tian S., Long J., Peng S., Xu L., Sun W., Chu H. (2020). Remarkable phosphate recovery from wastewater by a novel Ca/Fe composite: Synergistic effects of crystal structure and abundant oxygen-vacancies. Chemosphere.

[42] Jing L., Fu H., Wang B., Wang D., Xin B., Li S., Sun J. (2006). Effects of Sn dopant on the photoinduced charge property and photocatalytic activity of TiO_2_ nanoparticles. Appl. Catal. B Environ..

