# Supplementary material for: Enhanced Removal of Malachite Green Using Calcium-Functionalized Magnetic Biochar
Source: Int J Environ Res Public Health. 2022 Mar 10;19(6):3247. doi: 10.3390/ijerph19063247 (PMC8954663; doi:10.3390/ijerph19063247)
Supplement: Supplementary file 1 [file ijerph-19-03247-s001.zip › ijerph-1609401-supplementary.pdf]

Enhanced removal of malachite green using calcium-functionalized magnetic biochar

Pengjie Wang[1],·Wei Chen[1*],·Rui Zhang[1],·Yanfeng Xing[1*]

[1] Heilongjiang Ecological Environment Monitoring Center, Harbin, China

*** Corresponding authors.**

Wei Chen *E-ma*il: hljhjjc@yeah.net

*** Corresponding authors.**

Yanfeng Xing *E-ma*il: hljair@yeah.net

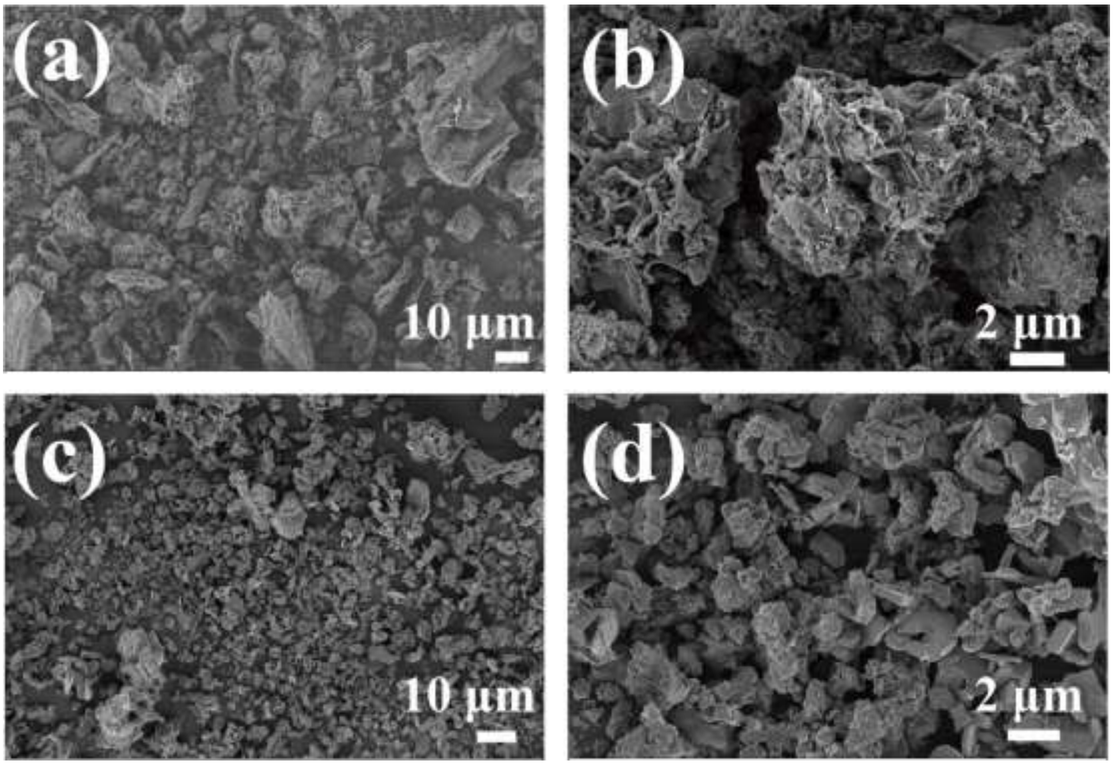

Figure. S1. SEM images of MBC (a, b) and Ca/MBC (c, d) .

	SSA [a] (m^2/g)	PV [b] (cm^3/g)	PS [c] (nm)
**MBC**	9.555	0.0214	12.684
**Ca/MBC**	2.779	0.0027	4.677

Table S1 Surface Area, Pore Volume, and Pore Size of MBC and Ca/MBC

a Specific surface area; b Pore Volume; c Pore Size.

Table S2. Adsorption kinetic fitting parameters.

	**Pseudo-first order**			**Pseudo-second order**			**Elovich**		
	q_3	k_1	R_1^2	q_2	k_2	R_2^2	α	β	R_3^2
**Ca/MBC**	1976.93	0.822	0.997	2027.207	2.53×10^{-4}	0.999	3.83×10^{24}	0.029	0.997

Table S3. Adsorption isotherm fitting parameters.

	Langmuir			Freundlich		
	q_1	K_L	R_1^2	K_F	$1/n$	R_2^2
**Ca/MBC**	12187.57	4.404×10^{-4}	0.997	13.198	0.811	0.974
